# Workflows and Laboratory Cost for Removable Digital Complete Denture: Two Case Reports with and without Existing Denture

**DOI:** 10.1155/2024/1564153

**Published:** 2024-02-03

**Authors:** Siraphob Techapiroontong, Nareudee Limpuangthip, Wisarut Prawatvatchara, Duangporn Yongyosrungrueng, Issarapong Kaewkamnerdpong

**Affiliations:** ^1^Department of Prosthodontics, Faculty of Dentistry, Chulalongkorn University, Bangkok, Thailand; ^2^Faculty of Dentistry, Chulalongkorn University, Bangkok, Thailand; ^3^Department of Community Dentistry, Faculty of Dentistry, Chulalongkorn University, Bangkok, Thailand

## Abstract

The integration of digital dentistry in the fabrication of complete dentures (CDs) has been facilitated through the use of intraoral scanners and computer-aided design and manufacturing (CAD/CAM). However, the financial implications associated with the fabrication of digital CDs have been rarely explored. This study is aimed at presenting two different approaches to CD fabrication, combining conventional with digital techniques, and comparing the total cost of fabrication. The first case involved a 70-year-old woman without existing denture, while the second case involved a 97-year-old woman with inadequate retention and stability of CDs in both jaws. In the first patient, who lacked information about her old denture, the first approach was employed, utilizing milling technology for denture processing. The second patient, who already had an existing denture, underwent the second approach, which employed printing technology for denture processing. CAD/CAM replicas of the existing dentures were used for the final impression, bite registration, and as a guide for tooth arrangement. Two digital protocols and laboratory cost in CD fabrication have been proposed. The relatively high cost of CAD/CAM CDs restricts the widespread of digital technology in CD fabrication.

## 1. Introduction

Digital technology has been used in removable complete denture (CD) fabrication to simplify clinical and laboratory procedures, reduce the number of clinical appointments [1, 2], and minimize errors resulting from conventional denture fabrication techniques and materials. Digital approaches comprise data acquisition using an intraoral scanner (IOS) and 3-dimensional (3D) scanning machine, computer-aided design (CAD), and computer-aided manufacturing (CAM) technology [3, 4]. The digital CD processing technologies consist of milling, which utilizes a subtractive technique from a prepolymerized PMMA disc using a milling machine [4], and 3D printing or rapid prototyping, which utilizes layer-by-layer fabrication techniques [3].

Digital technology has been incorporated into or replaced various conventional techniques used in CD fabrication to simplify the clinical and laboratory processes and reduce errors. The workflows and techniques vary depending on the available materials, instruments, and machines. Digital CDs can be fabricated either as a new denture without previous information [1, 5], or using an existing denture as a reference [6]. Various clinical settings have reported the cost-saving benefits when using digital technology in CD fabrication, including dental schools in the United States of America [7] and Switzerland [8, 9]. However, the financial implications of adopting digital technology for CD fabrication in low- to middle-income countries have rarely been explored.

The purpose of this case report was to describe two different approaches for CD fabrication that combined conventional and digital techniques. The first approach was employed in a patient who did not have old denture information and utilized milling technology for denture fabrication. The second approach was used in a patient who had an existing denture as a reference and utilized printing technology for denture fabrication. The laboratory cost for prosthesis fabrication was also compared between the two approaches and with the conventional procedure.

## 2. Case Presentation

### 2.1. Case 1

A 70-year-old woman presented at the Prosthodontic Clinic, Faculty of Dentistry, Chulalongkorn University, with a chief complaint of poor masticatory function. Her clinical examination found no soft tissue ulcers or trauma, and her edentulous condition indicated severe bone resorption and a significant loss of vertical dimension.

**Figure 1:**
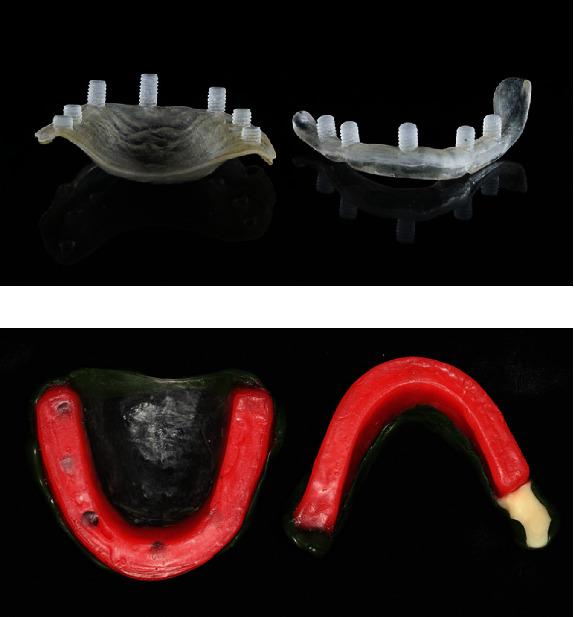
(a) Printed custom impression trays with screw-like patterns to serve as a guide for the occlusion rim wax-up. (b) Custom impression trays with a wax occlusion rim after adding autopolymerized acrylic resin on the retromolar pad area and border molding.

During the first visit, the maxillary and mandibular edentulous arches were scanned using an intraoral scanner (PANDA P2; Freqty, Zhejiang, China) to create two stereolithography (STL) files. However, the intraoral scanner was unable to capture the entire right retromolar pad due to the presence of flabby tissue. The files were imported to the CAD software (3Shape Dental System; 3Shape, Copenhagen, Denmark) to create digital study models for custom tray design. Two custom impression trays with a baseplate and occlusion rim design were created to use for the final impression and bite registration. The tray border was shortened by 2 mm from the tissue border to allow for border molding material. The potential occlusal plane level of the mandibular arch was defined as the midpoint of the retromolar pad height. Five to six screw-like patterns were placed along the cameo surface of the custom trays as a guide for occlusion rim placement along the alveolar ridge, with an average tooth height of 4 mm and 10 mm for the posterior and anterior teeth, respectively (Figure 1(a)). The custom impression trays were printed with clear-color photopolymer (Biocompatible Clear MED610™; Stratasys, Rehovot, Israel) using a 3D printing machine (Form3; Formlabs, Massachusetts, USA). The wax occlusion rims were added on the custom impression trays using the screw-like patterns as a guide for their height and buccolingual position.

**Figure 2:**
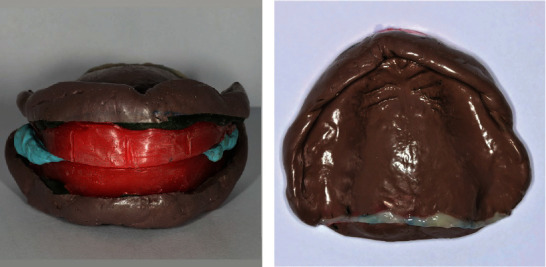
(a) Maxillomandibular relationship record silicone on the occlusion rim with polysulfide impression. (b) Flowable composite placed on the polysulfide final impression to transfer the PPS.

At the second clinical appointment, the custom impression trays were tried in the patient's mouth. The right mandibular custom tray was extended with autopolymerized acrylic resin (UNIFAST™ Trad; GC America Inc., Illinois, USA) to cover the unscannable retromolar pad (Figure 1(b)). The wax rims on the custom trays were adjusted to the determined occlusal vertical dimension and esthetics, including the smile line, lip support, and buccal corridor. The anterior tooth positioning indicators, including the dental midline and canine positions, were marked on the wax rims. Border molding was performed using a greenstick modeling plastic impression compound (Impression Compound; Kerr Corporation, California, USA). Definitive impressions of the maxillary and mandibular arches were made with rubber base (Impression Paste; S. S. White Group, Gloucester, UK), and the maxillomandibular relationship was recorded using silicone (Occlufast; Zhermack, Badia Polesine, Italy) at the predetermined vertical dimension (Figure 2(a)). The posterior palatal seal was identified by having the patient to vigorously say “ah” to locate the posterior limit of the posterior palatal seal, and the compressible area was identified using a ball burnisher to locate the posterior palatal seal area. The posterior palatal seal was marked using a water-soluble colored pencil and transferred to the final impression, and flowable composite resin (Filtek™ Supreme Flowable Restorative; 3M, Minnesota, USA) was directly applied onto the polymerized impression material without dental adhesive use (Figure 2(b)). The impressions and maxillomandibular relationship records were scanned using a 3D scanner to create the STL file for the virtual master cast. Virtual tooth arrangement and denture base design were performed by the CAD software following the impression's extension and border thickness.

**Figure 3:**
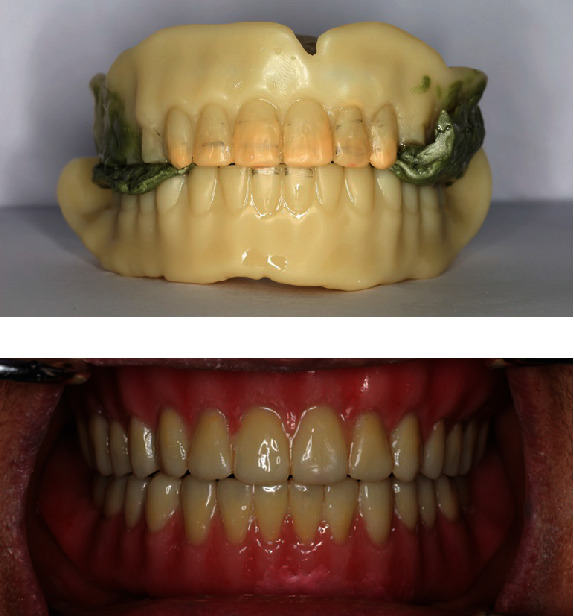
(a) Modifications done on the printed trial denture with the maxillomandibular relationship recorded with Aluwax. (b) Finished denture and delivery.

At the third clinical appointment, the printed trial dentures (NextDent Try-In; NextDent, Soesterberg, Netherlands) were inserted into the patient's mouth to verify tooth position, vertical dimension, and centric occlusion. Wax was added to the labial and incisal edges of the maxillary anterior teeth for recontouring. Because there was a discrepancy in the maxilla-mandibular relation, the occlusal surfaces of the maxillary posterior teeth were grinded with acrylic carbide burs and the maxilla-mandibular relation was rerecorded with Aluwax (Aluwax; ALUWAX DENTAL PRODUCTS CO., Michigan, USA) (Figure 3(a)). The trial dentures and maxillomandibular relationship record were then sent to the laboratory to rescan and redesign. The final dentures were designed by the CAD software, and the data sets were generated for the denture bases, tooth sockets, and denture teeth. When using CAM technology for denture fabrication, the denture base and teeth were printed separately using pink (NextDent Denture 3D+; NextDent, Soesterberg, Netherlands) and white photopolymer (NextDent C&B MFH; NextDent, Soesterberg, Netherlands) and were bonded together using an adhesive (Palabond®; Kulzer, Hanau, Germany). To enhance esthetics, the denture was stained with light-curing paste stains (Lite Art; SHOFU Dental, Kyoto, Japan). The final dentures were delivered at the fourth clinical appointment (Figure 3(b)).

### 2.2. Case 2

A dependent 97-year-old woman who needed a wheelchair presented with loose dentures on both jaws. The existing denture demonstrated unacceptable retention and stability and moderate attrition of the occluding surfaces of the artificial teeth with sliding and unstable occlusion. The new denture was fabricated using the technique of duplicating the existing dentures.

At the first clinical appointment, the existing dentures were scanned using a 3D desktop scanner, covering all internal and external surfaces, to generate the 3D data of the denture form in an STL file. The printed duplicate denture that functioned as a custom tray and bite registration was fabricated using clear-color photopolymer (Biocompatible Clear MED610™; Stratasys, Rehovot, Israel).

**Figure 4:**
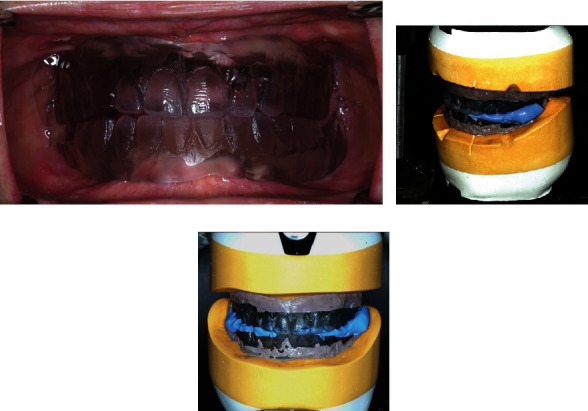
(a) Duplicate dentures tried in the patient's oral cavity. (b) Poured dental stone casts mounted on the articulator. (c) Notches made on the land area of the master casts.

At the second appointment, the duplicate dentures were tried in the patient's oral cavity (Figure 4(a)). These functioned as a custom tray, a maxillomandibular relationship record, and a guide for anterior tooth arrangement. After adjusting the border to provide at least a 2 mm space, border molding and functional impression procedures were performed using greenstick modeling compound and polysulfide impression material (Light Body Permlastic; Kerr Cooperation, California, USA). A putty-type polyvinylsiloxane (Silagum Putty; DMG, Hamburg, Germany) material was used to record the maxillomandibular relationship. The PPS was identified in the oral cavity with a ball burnisher using anatomical structures, i.e., the fovea palatini and maxillary notches, as references. The maxillary and mandibular final impressions were poured with type IV dental stone to fabricate the master casts, which were mounted on an articulator (Hanau™ Modular Articulator; Whip Mix, Kentucky, USA) (Figure 4(b)). Notches were made on the land area of the master casts as an index for overlapping the tissue surface and tooth position after removing the duplicated denture (Figure 4(c)). The outer surface of the duplicate dentures and land area of the master casts were scanned using the 3D desktop scanner. After removing the duplicate dentures, the posterior palatal seal was created on the maxillary stone cast using a steel round bur. The tissue surfaces of the master cast and notches on the land area were scanned. The tissue surface and tooth position overlapped through the notches to create a virtual master cast mounting in the CAD software.

**Figure 5:**
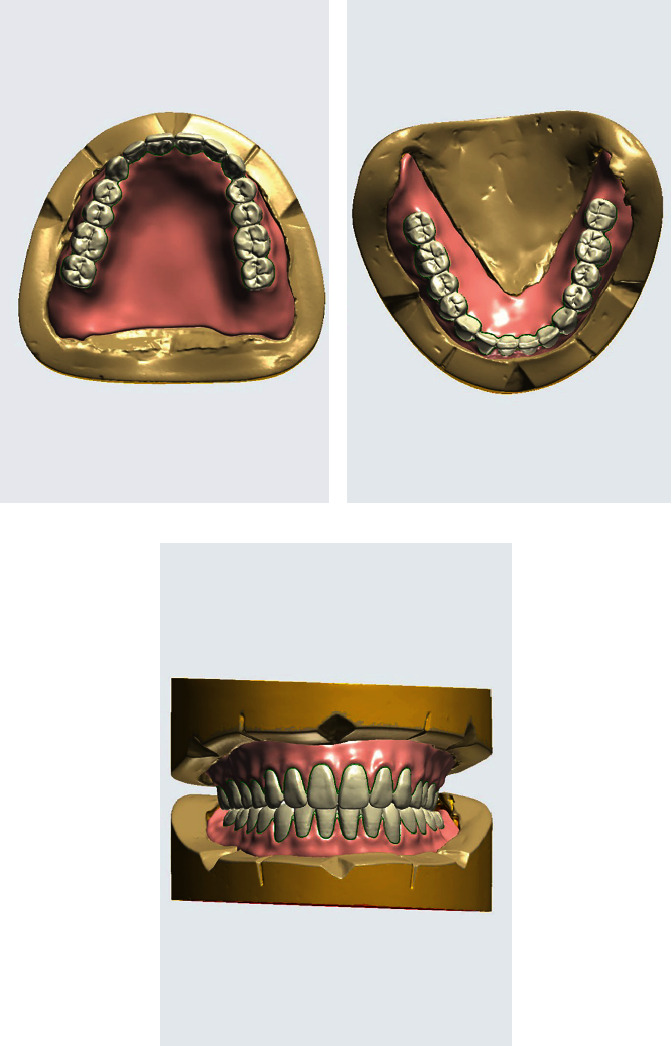
(a) Maxillary posterior tooth arrangement. (b) Mandibular posterior tooth arrangement. (c) Maxillomandibular relationship with no horizontal overlapping of the left maxillary and mandibular molar teeth.

**Figure 6:**
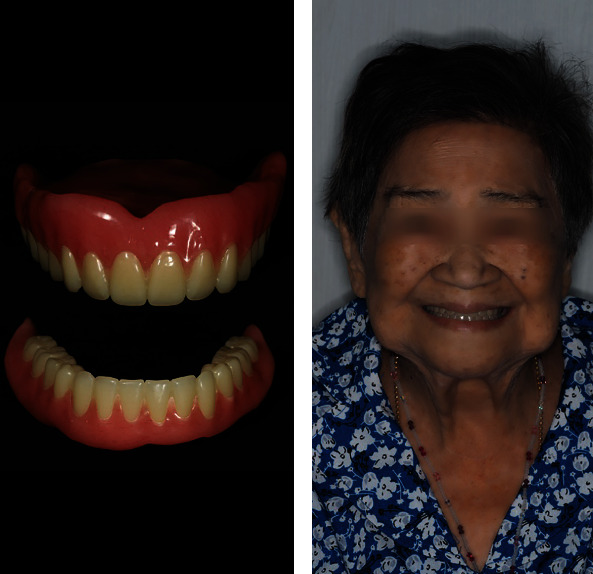
(a) Finished denture. (b) Denture delivery.

Tooth arrangement was performed virtually using the scanned duplicated denture as a reference. The dental midline and maxillary tooth positions were set according to the existing maxillary teeth, and the mandibular anterior teeth were arranged to provide optimal vertical and horizontal overlap with the maxillary teeth. The posterior anatomical teeth were initially positioned using the default tooth library that provides horizontal overlap between the maxillary and mandibular buccal cusps. The maxillary posterior teeth were then moved lingually to conform to the supporting residual ridge (Figures 5(a) and 5(b)), resulting in no horizontal overlap of the left maxillary and mandibular molar teeth (Figure 5(c)). The occlusal surfaces of the posterior teeth on both sides were adjusted into a nonanatomical tooth form. For denture processing, denture teeth (Vipi Trilux; Dentsply Sirona, North Carolina, USA) and base (T.S.M. Acetal Dental; Pressing Dental, San Marino, San Marino) were milled separately and bonded together with an adhesive and self-cured acrylic resin (Figures 6(a) and 6(b)). The high spot areas on the tissue surface were checked with pressure indicator paste, and the finished dentures were delivered.

Both patients were reappointed at 3 days, 1 week, and 1, 3, and 6 months after denture delivery. The laboratory costs for the two digital approaches were calculated based on the final dental laboratory invoice, compared with those of solely conventional procedures (Table 1). According to the laboratory company, the CD cost consisted of 40% material cost, 53% laboratory cost (encompassing manpower, processing machine, and related equipment), and 7% tax for both conventional and CAD/CAM dentures. The expenses for both digital denture techniques were approximately 10-fold higher compared with the conventional technique, primarily due to increased costs associated with materials and processing machines.

## 3. Discussion

The present study demonstrated two workflows for CD fabrication using digital technology. In this report, tooth arrangement was performed by virtually superimposing them on the occlusion rim or the existing denture to preserve the clinical information. This approach avoids the occlusion rim detachment required for arranging conventional teeth, which may cause tooth positioning errors. In accordance with previous studies [6, 10–12], using digital technology can reduce the number of clinical appointments required for CD fabrication, particularly in patients whose existing denture is used as a reference. Despite using digital technology, conventional border molding, final impression, and bite registration procedures are required. This is because the IOS cannot capture tissue and muscle movement and cannot be inserted into the mouth to capture the maxillomandibular relationship while maintaining baseplate stability.

The present study demonstrated various alternative techniques when using digital technology in CD fabrication. Screw-like patterns on the custom trays assist in establishing the occlusion rim and conform it to the underlying residual ridge, especially when an actual preliminary cast has not been fabricated. Virtual master models can be created by scanning either the impression or the poured stone cast. Pouring the stone cast becomes necessary when a 3D scanner is not available, and the impression material, such as polysulfide, needs to be poured within a limited time. Notches created on the land area of the stone master casts aided in transferring the maxillomandibular relationship from the conventional to the virtual articulator. To transfer the posterior palatal seal from the patient's oral cavity to the virtual master cast, previous studies have reported carving the outline and depth in the CAD software based on clinical measurement [1] or the marked impression [13, 14]. Our study demonstrated a technique that uses flowable composite resin to transfer the outline and depth of the posterior palatal seal to reduce errors that may arise from a technician carving it. The techniques presented in this study help reduce errors when transferring the information from the clinic to the laboratory technician.

There are some potential concerns when using digital technology in CD fabrication. Taking a digital impression with an IOS can be difficult due to anatomical limitations [5], especially in cases with a flabby narrow residual ridge and large tongue, resulting in incomplete data capture. Moreover, the virtual tooth arrangement based on the default settings can result in inappropriate tooth position and occlusion, including incisal contact of the anterior teeth and positioning the maxillary posterior teeth too buccally. To achieve optimal function and esthetics, further adjustments may be necessary, including removing the lingual contour of the maxillary anterior teeth to prevent anterior tooth contact in centric occlusion and moving the maxillary posterior teeth lingually to avoid cheek biting caused by an out-of-ridge placement. Adjusting the printed trial denture requires griding and the addition of wax or composite resin. An additional CAD/CAM trial denture may be requested by the dentist or the patient for each new trial, in contrast to the conventional approach where the existing wax baseplate and artificially teeth can be reused. This may result in added cost to the patient.

Previous studies have assessed the cost of fabricating CAD/CAM CDs, including laboratory and dental treatment costs [8, 9]. In our dental school, however, the treatment cost for the CAD/CAM CDs has not yet been established because the treatment has not yet become a routine postgraduate practice due to the high laboratory cost. Therefore, this report considered only the laboratory cost, which is substantially higher for the CAD/CAM approach compared with the conventional technique. In addition, the initial investment for digital equipment in the clinic, including the IOS and 3D scanner, is approximately 1,000,000 Thai baht/~30,000 USD (as of January 2024). The manpower needed for the conventional and digital approaches is similar but requires different skill sets. In contrast to previous studies in high-income countries [7–9], the present case report revealed that CAD/CAM CDs may not always be a cost beneficial solution for edentulous patients due to high costs compared with the conventional procedure. In this report, the two patients preferred the conventional rather than the digital approaches if they had to pay for the treatment themselves.

The present workflows demonstrated the alternative workflows for removable CD fabrication that combined the conventional and digital techniques. The advantages and points of concern when using digital technology in CD fabrication have been demonstrated which may be useful for dentists and dental technicians. However, further studies are required to determine the cost benefit of integrating digital technology into routine CD fabrication procedures.

## 4. Conclusion

The present case report demonstrated two different approaches for CD fabrication that combined conventional and digital techniques. The potential advantages and concerns of using digital technology were discussed. Although there are several benefits of integrating digital into conventional techniques, the higher laboratory costs associated with CAD/CAM CDs may limit its use in some dental practices.

## Figures and Tables

**Table 1:** Laboratory cost in THB (35.63 THB =1 USD as of January 2024) used for printing and milling compared with conventional approach.

Prosthesis	Conventional	Digital	
		Case 1 (without existing denture)	Case 2 (with existing denture)
Custom trays	120 (800 for duplicated denture)	2,000 (print)	2,000 (print)
Baseplate and occlusion rim	240		
Trial denture		2,000	None
Final prosthesis		(Print)	(Mill)
(i) Denture base	700	4,000	6,000
(ii) Denture teeth	300–1,200	5,600 (400 per tooth)	8,400 (600 per tooth)
(iii) Staining	None	2,800 (100 per tooth)	None
Total lab cost (per piece) (per pair)	1,360–2,700 2,720–5,400 (~80–160 USD)	16,400 32,800 (~960 USD)	16,400 32,800 (~960 USD)

## Data Availability

The raw data of this study will be provided upon request to the corresponding author.
